# Photodynamic Antimicrobial Activity of a Novel 5,10,15,20-Tetrakis (4-Ethylphenyl) Porphyrin against Clinically Important Bacteria

**DOI:** 10.3390/ph16081059

**Published:** 2023-07-26

**Authors:** Fabián Espitia-Almeida, Roger Valle-Molinares, Elkin Navarro Quiroz, Leonardo C. Pacheco-Londoño, Nataly J. Galán-Freyle

**Affiliations:** 1 Life Science Research Center, Universidad Simón Bolívar, Barranquilla 080002, Colombianataly.galan@unisimon.edu.co (N.J.G.-F.); 2 Faculty of Basic and Biomedical Sciences, Universidad Simón Bolívar, Barranquilla 080002, Colombia; 3 Faculty of Basic Sciences, Biology Program, Universidad del Atlántico, Puerto Colombia 081001, Colombia

**Keywords:** photodynamic, antimicrobial activity, porphyrin, *Pseudomonas aeruginosa*, MRSA, *Staphylococcus aureus*, photophysical properties

## Abstract

The growing emergence of microbes resistant to commercially available antibiotic therapies poses a threat to healthcare systems worldwide. Multiple factors have been associated with the increasing incidence of hospital-acquired infections caused by antibiotic-resistant pathogens, including the indiscriminate use of broad-spectrum antibiotics, the massive application of antibiotics in hospitals as a prophylactic measure, self-medication, and nonadherence to pharmacological therapies by patients. In this study, we developed a novel treatment to mitigate the impact of microbial resistance. We synthesized a benzoporphyrin derivative, 5,10,15,20-tetrakis (4-ethylphenyl) porphyrin (TEtPP), with a reaction yield close to 50%. TEtPP exhibited excellent photophysical properties (Φ_f_ = 0.12 ± 0.04 and Φ_Δ_ = 0.81 ± 0.23) and was thereby assessed as a potential agent for antibacterial photodynamic therapy. The photophysical properties of the synthesized porphyrin derivative were correlated with the assayed antimicrobial activity. TEtPP showed higher activity against the MRSA strain under irradiation than in the absence of irradiation (minimum inhibitory concentration (MIC) = 69.42 µg/mL vs. MIC = 109.30 µg/mL, *p <* 0.0001). Similar behavior was observed against *P. aeruginosa* (irradiated MIC = 54.71 µg/mL vs. nonirradiated MIC = 402.90 µg/mL, *p <* 0.0001). TEtPP exhibited high activity against *S. aureus* in both the irradiated and nonirradiated assays (MIC = 67.68 µg/mL vs. MIC = 58.26 µg/mL, *p =* 0.87).

## 1. Introduction

The development of antibiotics has been a milestone in clinical practice for managing and controlling infectious diseases. Antibiotics have saved millions of lives, contributed considerably to the advancement of different fields of medicine and pharmacology (such as organ transplantation, the survival of premature neonates, and surgery), and have generally increased human life expectancy [1]. However, the increasing emergence of resistant bacteria has reduced the effectiveness of antibiotics [2,3].

Resistance is defined as the ability of bacteria to survive exposure to antibiotics at concentrations that would typically cause bacterial death. Antibiotic resistance has become one of the determining conditions for the increase in morbidity and mortality rates worldwide, the appearance of diseases associated with health care, and high hospitalization costs [1]. There has been a growing emergence of bacterial strains, such as *P. aeruginosa* and *S. aureus*, that are resistant to broad-spectrum antibiotics, such as carbapenems, quinolones, aminoglycosides, and cyclins [4]. Multiple factors have been associated with the appearance of this resistance, among which the most relevant have been identified in the literature as the indiscriminate use of broad-spectrum antibiotics, the massive application of antibiotics in hospitals as a prophylactic measure, self-medication, and lack of adherence to pharmacological therapies by patients [1,3]. These resistant bacteria are responsible for a variety of infections, ranging from skin and soft tissue infections to invasive infections, that can even lead to the death of the patient. An example is the methicillin-resistant *S. aureus* strain (MRSA), which has been reported as the cause of approximately 70% of infections in Intensive Care Units in countries such as Colombia [5].

As bacteria are developing increasingly efficient mechanisms of antibiotic resistance [6], more effective and less toxic chemical agents urgently need to be developed. To this end, medicinal chemistry provides a plausible means of designing and preparing novel synthetic derivatives of different families of compounds; evaluating the biological properties of these derivatives; establishing the degree of efficacy of these derivatives at different research scales (in vitro, ex vivo, and in vivo) against biological entities of interest, such as bacteria, fungi, viruses, parasites, and cancer cells; establishing possible relationships between chemical structure and biological activity (QSAR analysis); and facilitating the development of novel drugs [7,8,9,10].

The use of porphyrin derivatives in conjunction with antimicrobial photodynamic therapy (APDT) has been one of the most commonly used strategies in the search for bioactive agents. Promising results have led to the development of successful treatments in different areas of medicine [5,6,7,8,9,10]. Therapies based on light and laser sources are being used as alternative means of diagnosing and treating diseases [11,12]. Photodynamic therapy (PDT) has proven to be an effective and highly selective treatment for diseases such as cancer, skin diseases, and various types of infection [10,13,14]. Compared to traditional antibacterial methods, PDT has been making procedures increasingly effective with fewer side effects, because it has been demonstrated that resistant cells do not form under repeated photosensitization [15].

Many porphyrin-like compounds have been described as potential photosensitizers for use in APDT against bacteria [16]. In particular, aromatic substituents, such as amphiphilic long side chains incorporated into benzoporphyrins [17], facilitate the entry of compounds into a target cell membrane and accumulation in the cytosol, where subsequent photoactivation by visible light causes singlet-oxygen-mediated cell death [18,19,20]. In general, functionalizing hydrophilic porphyrins with long hydrophobic side chains is expected to make the lipid bilayer in cell membranes more permeable to porphyrins [19,21].

Many compounds have been identified as potential photosensitizers for use in APDT against bacteria [16]. In particular, heterocyclic aromatic compounds, such as porphyrins, are ubiquitous compounds in nature and participate in vital biochemical processes, such as oxygen transport and photosynthesis [18]. The exceptional physicochemical and photophysical properties of porphyrins result in a wide spectrum of uses [22], such as artificial photosynthesis [23], oxidative catalysis [24], the production of nonlinear optical sensors [25], the fabrication of nanomaterials for photodynamic cancer therapy, and (recently) the photoinactivation of microorganisms [26]. This diversity of uses of the porphyrin ring has aroused the interest of organic chemists in synthesizing derivatives that exhibit chemical, physical, and electronic properties that increase the effectiveness of porphyrins for the aforementioned applications [18].

In this study, we developed a novel metal-free porphyrin using synthesis and characterization processes previously reported by our research team. Our aim was to evaluate the potential of this novel molecule as an alternative treatment for infections caused by *P. aeruginosa* and *S. aureus*. We found that the novel 5,10,15,20-tetrakis (4-ethylphenyl) porphyrin has a potent inhibitory effect at low concentrations on *P. aeruginosa* and MRSA using PDT and on a susceptible strain of *S. aureus* with and without the use of PDT.

## 2. Results and Discussion

### 2.1. Porphyrin Structural Characterization via UV-Vis

**Figure 1:**
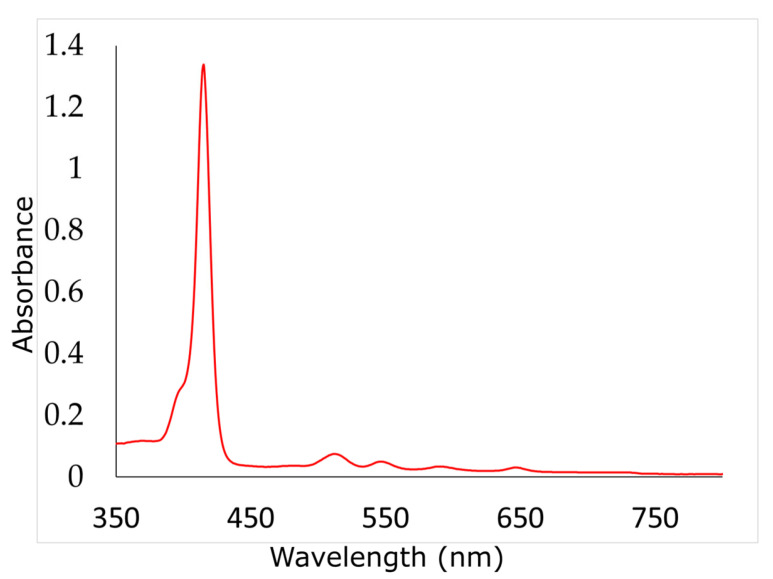
UV-Vis spectrum of the synthesized compound in ethyl acetate at pH = 7 obtained at room temperature.

Figure 1 shows the absorption spectrum of TEtPP, which exhibits the characteristic band pattern for metal-free porphyrins reported in the literature [27]. The Soret band with maximum absorption at 415 nm is associated with a_1u_(π)–eg∗(π) electronic transitions, and the four weaker bands (Q bands) at 512, 547, 590, and 646 nm are attributed to a_2u_(π)–eg∗(π) electronic transitions of the highly conjugated porphyrin ring [27]. Derivatization enables porphyrins to make electronic transitions from the highest occupied molecular orbital to the lowest unoccupied molecular orbital at wavelengths above 400 nm, modifying their photophysical, optical, and redox properties. Thus, porphyrins can be transformed into very useful chemical structures for designing and synthesizing novel compounds with potential application in medicinal chemistry and the biological sciences [28,29,30].

The aforementioned structural modifications provide the porphyrin molecule with a certain degree of tropism or selectivity toward biological entities of interest, such as bacteria, viruses, fungi, parasites, and tumor cells [7,31,32,33,34]. For almost three decades, medicinal chemistry studies on the development of novel drugs have been based on analyzing the chemical structure and biological activity of a molecule (QSARs—quantitative structure–activity relationships) to predict and optimize pharmacological activity [35,36].

Few studies have been conducted on how structural diversification of the porphyrin ring affects antibacterial activity. Studies have mainly focused on how the addition of metals can enhance the antibacterial activity of the porphyrin ring. The activity of the porphyrin ring against bacteria (such as those tested in this study) has been shown to depend on the type of substituent used (see, for example, Ooi et al., 2009 and Quintana et al., 2018) [31,37]. Therefore, it is important to incorporate substituents identified via QSAR into the base ring that can enhance activity against bacteria of clinical interest to mitigate resistance against available antibiotic therapies. Thus, we incorporated the substituent 4-ethylphenyl into the porphyrin ring to improve its base antibacterial activity. The present study needs to be complemented with cytotoxicity and genotoxicity analyses to demonstrate that the increase in antibacterial activity did not result from an increase in the cytotoxicity of porphyrin.

**Scheme 1:**
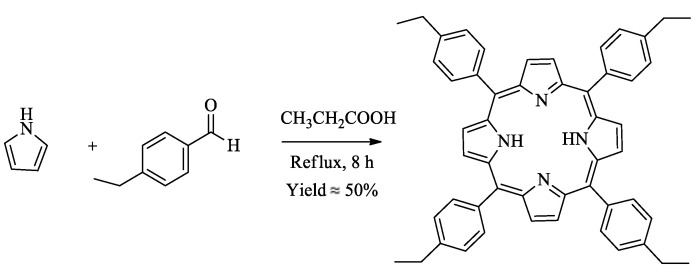
Chemical synthesis of 5,10,15,20-tetrakis (4-ethylphenyl) porphyrin (TEtPP).

The presence of the porphyrin fingerprint, Soret band, and four Q bands in the UV-Vis spectrum shows that the desired product was synthesized (Scheme 1).

### 2.2. FTIR Spectra

**Figure 2:**
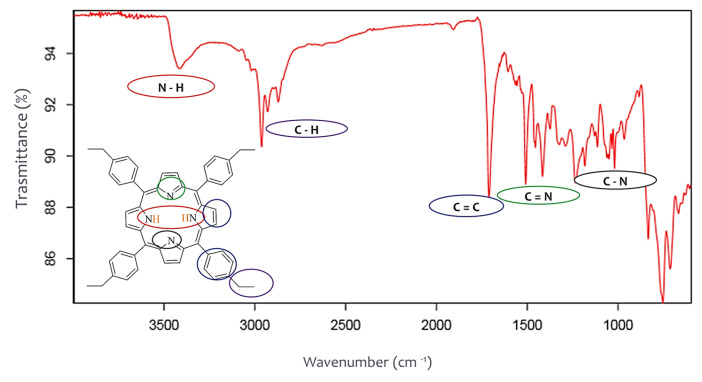
FT-IR spectrum.

The FT-IR spectrum (Figure 2) exhibited characteristic signals of the main functional groups in porphyrin compounds, including stretching of the N–H bond in the porphyrin ring at 3350 cm^−1^; stretching of the C–H bond for sp3 carbons at 2960 cm^−1^, as well as of C=C in the vicinity of 1470 cm^−1^, C=N at 1088 cm^−1^, C–N in the vicinity of 964 cm^−1^; and out-and-in-plane torsion vibrations for the N–H bond at 795 cm^−1^ [27].

### 2.3. Fluorescence Quantum Yield

**Figure 3:**
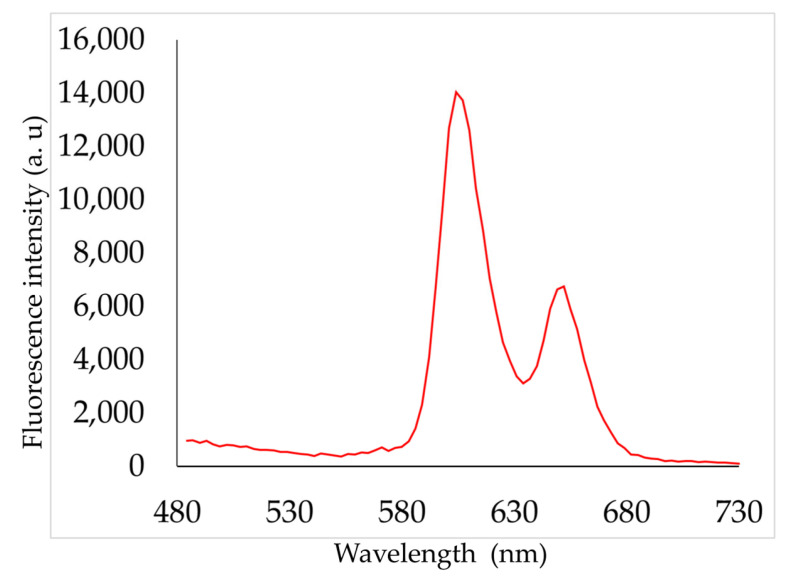
Emission spectra of TEtPP in ethyl acetate at pH = 7.0 obtained at room temperature. Spectra at 3.0 9 10^−5^ M concentration.

Figure 3 shows the fluorescence spectrum of TEtPP, which exhibits maximum emission peaks at 604 and 652 nm, evidencing photoluminescence in the blue region of the electromagnetic spectrum. The generation of these peaks is associated with changes in the excited states of the molecule from a minimum-energy state So (relaxed) to a maximum-energy state S1 (excited) [35]. The TEtPP molecule remains in S1 for a short time and subsequently switches to So, emitting fluorescent radiation characteristic of porphyrins [18]. The area under the corrected emission spectrum was used to calculate Φ_f_ as 0.12 ± 0.04, which was similar to the value previously reported by our working group [27]. In the literature, a low Φ_f_ for porphyrin compounds ensures a high production of singlet oxygen. Consequently, the performance of porphyrins is improved in some applications reported for this porphyrin system, especially in FDT, where it acts as a potential photosensitizing agent that can destroy cancer cells, viruses, bacteria, fungi, and parasites [10,13,14,36].

### 2.4. Singlet-Oxygen Quantum Yield

**Figure 4:**
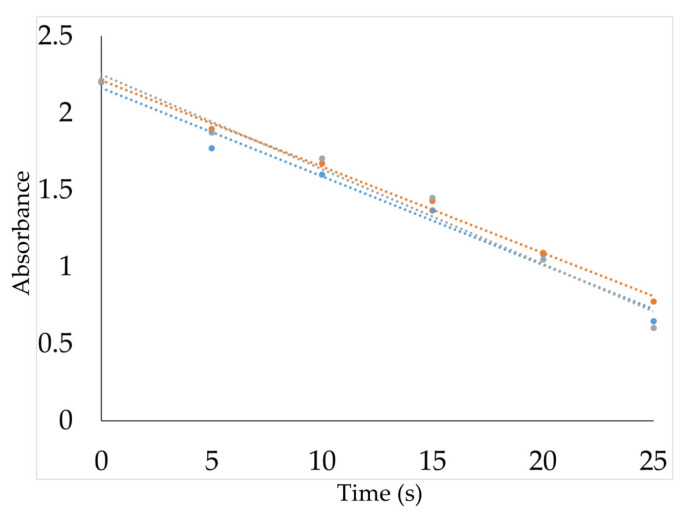
Determination Φ_Δ_ of TEtPP in Dimethylformamide at pH = 7 and room temperature.

The singlet-oxygen quantum yield was determined by preparing a 1,3-diphenylisobenzofuran (DPBF) solution with a measured absorbance of 2 ± 0.05 (λ = 410 nm). The degradation of this dye is directly proportional to the production of singlet oxygen. Figure 4 shows the degradation curve obtained by irradiating the sample solutions with monochromatic light with λ = 600 nm. We used this curve to determine the degradation constants (kd) of TEtPP and the H_2_TPP standard, which, in turn, were used to calculate Φ_Δ_ as 0.81 ± 0.23 in Dimethylformamide (DMF), which is similar to a previously reported value [27].

### 2.5. Photodynamic Antimicrobial Effect

**Figure 5:**
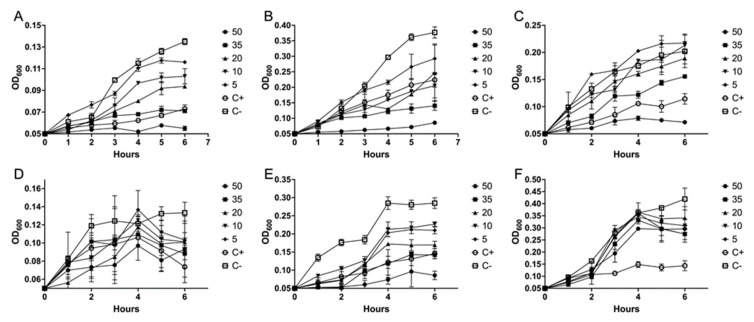
Growth curves of different bacteria (*P. aeruginosa*, *S. aureus*, and MRSA) treated using TEtPP with (**A**–**C**), respectively) and without (**D**–**F**), respectively) irradiation under visible light for 15 min.

Some porphyrinic compounds have been evaluated as photosensitizing agents against resistant and sensitive bacteria in the search for alternative strategies for mitigating the negative impact of bacterial resistance in health systems worldwide [31,37,38]. However, this search has not yielded the expected results. Therefore, the design and synthesis of novel porphyrin derivatives with groups or side chains that improve the photophysical and photodynamic properties of porphyrins represents an important topic for medicinal chemistry and the biological sciences. In this study, we developed a novel synthetic benzoporphyrin. We performed in vitro assays to evaluate the ability of the synthesized compound to inhibit the growth of three types of bacteria, one sensitive and two resistant, under visible light irradiation. We constructed growth curves for each test bacterium to assess the effects of irradiation and photosensitizer concentration on bacterial growth (Figure 5). The bacterial growth rate was retarded at all the evaluated photosensitizer concentrations, and this retardation became more prominent under irradiation.

**Figure 6:**
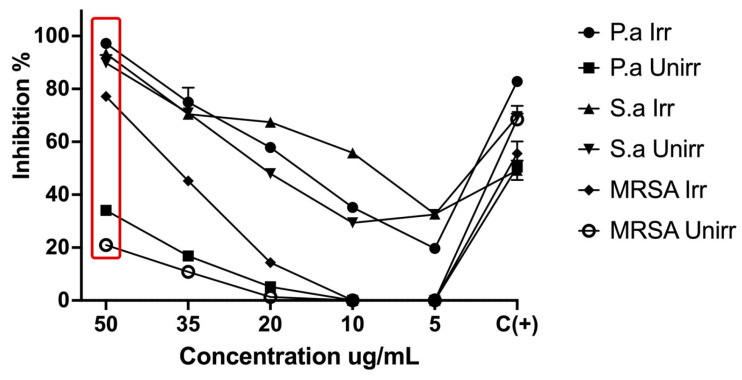
Calculated percent inhibition by TEtPP at different concentrations against the test bacteria with and without irradiation.

Similarly, an exponential growth phase was observed in the negative control (bacteria + culture medium in the absence of the photosensitizer) after 4 h of incubation for all strains. Thus, 4 h was used as the reference time to calculate the percent inhibition for each bacterium at each photosensitizer concentration. The inhibitory bacterial activity was found to increase with the photosensitizer concentration (Figure 6).

**Figure 7:**
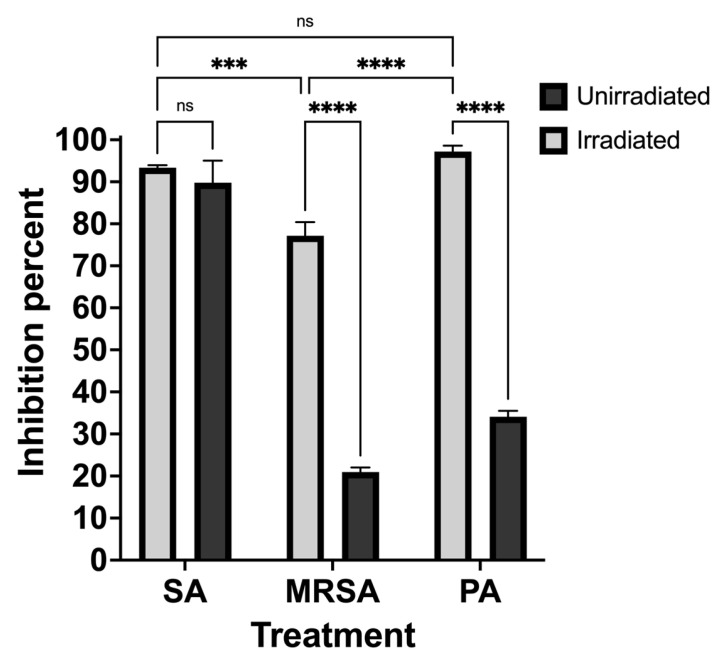
Photodynamic effect of 50 µg/mL TEtPP against *P. aeruginosa*, *S. aureus*, and MRSA with and without irradiation, where *** indicates a statistically significant result of *p <* 0.001 and **** indicates a statistically significant result of *p <* 0.0001.

More than 80% inhibition of *P. aeruginosa*, *S. aureus*, and MRSA was obtained following treatment with 50 µg/mL of photosensitizer both with and without irradiation. The wildtype strain of *S. aureus* exhibited the highest sensitivity to the inhibitory effect of porphyrin, which can be attributed to photoactivation of the porphyrin molecule under environmental light [13,39]. Molecular photoactivation has been reported to facilitate the production of small quantities of ROS without the use of special equipment to induce photoactivation [8]. It has been proposed in some studies that the photoactivation of molecules, such as those used in this study, occurs in different parts of the bacterial cell, depending on the ability of the photosensitizer to penetrate the bacterial cytoplasm [40]. The permeability of the photosensitizer could be affected by the antibacterial resistance mechanism of the microorganism. Thus, the different resistance mechanisms of *P. aeruginosa* and MRSA from that of the wildtype strain of *S. aureus* could explain why the inhibitory effect of the photosensitizer on these two strains was found to depend on photoactivation for *P. aeruginosa* and MRSA but not for *S. aureus* (Figure 7).

The theoretical minimum inhibitory concentration (MIC) was consistent with the determined inhibition percentages (Table 1). The MICs for *P. aeruginosa* and MRSA were lower for the treatments with irradiation than those without irradiation, whereas the MICs for the treatments of the wild strain of *S. aureus* with and without irradiation were very close, confirming the susceptibility of the wild strain of *S. aureus* to treatment with the novel photosensitizer. Our results suggest that the proposed treatment has high therapeutic potential for the treatment of infections caused by the three test strains, particularly susceptible strains of *S. aureus*, which are frequently associated with skin infections. Thus, the proposed treatment would facilitate the application of photodynamic therapy. The experimental data show that TEtPP could inhibit the growth of *S. aureus* without photoactivation using special equipment.

The results presented in Table 1 show considerably lower MICs for irradiated treatments against Gram-positive and Gram-negative bacteria than those reported in the literature, particularly against *P. aeruginosa* (4-TPyPor systems, MIC = 77.0 µg/mL; 4-PtTPyPor, MIC = 170.0 µg/mL) and *S. aureus* (4-TPyPor systems, MIC = 154.0 µg/mL; 4-PtTPyPor, MIC = 340.0 µg/mL) [31]. The methicillin-resistant mutant strain of *S. aureus* (MRSA) was found to be sensitive to treatment with TEtPP (69.42 µg/mL), which is a useful result considering that this bacterium is the cause of many deaths in hospitalized patients in Colombia, as well as worldwide [41,42]. Our results show that both Gram-positive and Gram-negative bacterial strains were inhibited by TEtPP, of which *P. aeruginosa* was the most strongly affected, as evidenced by the fact that it had the lowest MIC (54.71 µg/mL). This apparent contradiction may result from the structural diversity of the photosensitizers and different morphological characteristics of the bacteria used in different studies [43,44,45].

The mechanism of the death of bacteria after exposure to photosensitizers at different concentrations and irradiation with visible light is not known [46]. The antimicrobial activity of porphyrins is frequently attributed to their chemical properties, such as their ability to transfer electrons, catalyze the reactions of some enzymes (peroxidases, catalases, and oxidases), absorb photons and produce ROS, and break down the lipids in cell membranes [45]. However, one inhibition mechanism of bacterial growth that has been little studied to date is the binding of porphyrins to hemoproteins, where the resulting malfunction of these proteins affects bacterial metabolism [47,48]. This inhibition mechanism should be studied very carefully because there are very similar metabolic pathways in bacteria and mammals that require the intervention of heme groups, which suggests a possible cytotoxic effect in eukaryotes. Some studies have shown that the antibacterial activity of metal-free porphyrins is enhanced upon irradiation with visible light. However, the antimicrobial activity of a mixture of metal-free porphyrins and heme groups was also shown to be enhanced in the absence of light, especially against Gram-positive bacteria [45].

Another hypothetical mechanism of action for porphyrins is associated with the amphipathic nature of these molecules, which facilitates diffusion through the cell membrane (phagocytosis) and accumulation in the cytosol [20]. Subsequent irradiation with visible light in the presence of ambient triplet-molecular oxygen (^3^O_2_) enables porphyrins to absorb energy and transition from a relaxed base state So to an excited triplet state S1. Porphyrins in the S1 state can interact with ^3^O_2_, and the transfer of the previously absorbed energy induces ^3^O_2_ to transition to a singlet state (^1^O_2_) and porphyrins to return to a relaxed base state So. The highly oxidizing ^1^O_2_ species can inflict considerable damage on bacteria at the level of the cell membrane, cell organelles, structural and functional proteins, nucleic acids, and other vital bacterial components [19,20,36].

Evidence for the aforementioned mechanisms comes from the ability of TEtPP to produce singlet oxygen (Figure 4) and to remain for a sufficiently long period of time in the excited triplet state (*τ* > 1 µs) to interact with molecular oxygen in the environment. These results confirm the efficient action of porphyrins upon irradiation with visible light and the reduction in the phototoxic capacity of porphyrins in the dark.

## 3. Materials and Methods

### 3.1. Synthesis and Characterization of Porphyrins

The porphyrin (TEtPPs) was synthesized according to procedures previously standardized by Dr. Espitia-Almeida in his Ph.D. thesis [49]. Briefly, equimolar quantities of p-ethyl benzaldehyde and pyrrole were refluxed for 8 h in 80 mL of propionic acid (Scheme 1). The efficiency of product formation was close to 50%, as determined via thin layer chromatography. The product was recovered from the reaction mixture by adding 80 mL of cold methanol, followed by gravity filtering through Whatman No. 1 filter paper with a nominal pore size between 8 and 12 µm, yielding a crystalline compound with an intense purple color. The obtained compound was purified via column chromatography using chloroform–ethyl acetate (9:1) as the mobile phase and Millipore silica gel 60 F_254_ (0.063–0.200 mm) as the stationary phase. Finally, the synthesized compound was structurally characterized via UV-Vis, FT-IR, and fluorescence spectroscopy. All reagents were provided by Merck, Rahway, NJ, USA.

### 3.2. Photophysical Properties

The fluorescence quantum yield (Φ_f_) of TEtPP was determined using the steady-state comparative method [34]. The area under the corrected emission spectrum of a fluorescein standard dissolved in water was compared with that of TEtPP dissolved in ethyl acetate at pH = 7.0. Emission spectra were obtained by exciting fluorescein at λ = 493 nm and TEtPP at λ = 415 nm. The calculated Φ_f_ values were fitted using the refractive indices of the solvents (ɳ_ethyl acetate_ = 1.37 and ɳ_water_ = 1.33) in Equation (1). Assays were carried out in triplicate.

(1) $$\Phi_{f-TEtPP}=\Phi_{f-fluorescein}\times \frac{F_{TEtPP}*\lambda_{Fluorescein}}{F_{fluorescein}*\lambda_{TEtPP}}\times (\frac{{ɳ}^{2}\;{}_{water}}{{ɳ}^{2}\;{}_{ethyl\;acetate}})$$

In the equation above, F_TEtPP_ and F_Fluorescein_ denote the areas under the corrected emission spectra of TEtPP and the fluorescence standard, respectively; λ_TEtPP_ and λ_Fluorescein_ denote the excitation wavelengths of TEtPP and the fluorescence standard, respectively; ɳ_*ethyl acetate*_ and ɳ_water_ denote the refraction indexes of the solvents of ethyl acetate and water, respectively; and Φ_f_ denotes the fluorescence quantum yield of fluorescein.

The relative method was used to calculate the singlet-oxygen quantum yield (Φ_Δ_) of TEtPP using 1,3-diphenyl dibenzofuran (DPBF) as a singlet-oxygen trap and 5,10,15,20-(tetraphenyl) porphyrin (H_2_TPP) as a standard (Φ_Δ_ = 0.64 in DMF). A 1 × 10^−9^ M solution of TEtPP in DMF was used. The quantum yield was calculated using Equation (2). The assays were carried out in triplicate.

(2) $$\Phi_{\Delta_{\mathrm{TEtPP}}}=\Phi_{\Delta_{H_{2}\mathrm{TPP}}}\times \frac{W_{\mathrm{TEtPP}}}{W_{H_{2}\mathrm{TPP}}}$$

In the equation above, Φ_∆_ H_2_TPP denotes the singlet-oxygen quantum yield of the standard and W_TEtPP_ and W_H2TPP_ denote the photobleaching rates of TEtPP and H_2_TPP under irradiation, respectively.

UV-Vis and fluorescence spectra were obtained at room temperature and pH = 7.0 using a CLARIO Star Plus and a 1 mL quartz cell.

### 3.3. Strain and Growth Conditions

Three clinically important bacteria (*Pseudomonas aeruginosa* ATCC 15692, *Staphylococcus aureus* ATCC 259233, and methicillin-resistant *Staphylococcus aureus* ATCC 33591) were treated with a 5,10,15,20-tetrakis (4-ethylphenyl) porphyrin photosensitizer.

We used the following procedure to determine the kinetic growth parameters of the strains used in this study. A sterile inoculation loop was used to deposit a small portion of frost containing the cryopreserved strains (−86 °C) in a test tube containing 5 mL of nutrient broth. The mixture was incubated at 37 °C under shaking overnight. Subsequently, the absorbance of the mixture was measured at 600 nm using a spectrophotometer. The appropriate volume of the inoculum was determined using the dilution formula (V_1_ × C_1_ = V_2_ × C_2_). This volume was withdrawn from the test tube and added to a new test tube containing 5 mL of sterile nutrient broth to initiate growth in a culture with an optical density close to 0.05. The new culture was incubated at 37 °C, and the increase in the absorbance was recorded at hourly intervals until the stationary phase of bacterial growth was initiated. The growth curve data were used to identify a start time for all experiments corresponding to the middle of the exponential phase of growth, where the culture had an absorbance equivalent to the 0.5 standard on the McFarland scale (1.5 × 10^8^ cells/mL) [50].

### 3.4. Photodynamic Antimicrobial Therapy

A total of 1 mg/mL of dimethyl sulfoxide (DMSO) photosensitizer stock solution was used to prepare five working solutions of different concentrations (100, 70, 40, 20, and 10 µg/mL) using PBS at pH = 7. Next, bacteria in the middle of the exponential phase of growth were diluted to ~105 CFU/mL in PBS at pH = 7. Aliquots (50 μL) of the diluted solution were delivered to a 96-well plate and mixed with 50 μL of the treatment solution to achieve a final range of concentrations of 50, 35, 20, 10, and 5 µg/mL. Each treatment (corresponding to a specific photosensitizer concentration for a bacterial strain) was performed in triplicate under irradiation for 25 min using a vertical array of blue LEDs with emission wavelengths in the 420–450 nm range to achieve a light intensity of 80 J/cm^2^. Figure 4 shows that the synthesized compounds produced the highest levels of singlet oxygen in 25 min, which was therefore selected as the irradiation period. The optical density of the solutions was read, and the net growth was determined as the difference between OD_600_ at hourly intervals and time zero.

The inhibition percentage was calculated using Equation (3):

(3) $$\%I=\frac{{{OD}_{NC}-OD}_{T}}{{OD}_{T}}\times 100$$

where OD_NC_: the absorbance of the negative control, and OD_T_: absorbance of the treated sample.

Three controls were used to verify that the inhibitory effect was caused by the photochemical activation of porphyrin molecules: (i) an irradiation control consisting of the photosensitizer, test strain, and nutrient broth with no exposure to visible light, (ii) a negative control consisting of the test strain and nutrient broth (0% inhibition), and (iii) a positive control consisting of the test strain, nutrient broth, and gentamicin (100% inhibition).

The MICs were calculated via nonlinear regression, where the experimental data were fitted via a modified Gompertz equation using GraphPad Prism 9.3.1.

### 3.5. Statistical Analysis

All experiments were performed in triplicate. The experimental data were analyzed using GraphPad Prism 9.3.1. The statistical significance between the irradiated and nonirradiated porphyrin treatments for each bacterium of clinical interest was determined using two-way ANOVA and a Tukey’s test for multiple comparisons at a 95% confidence level. Differences were considered significant at *p <* 0.05.

## 4. Conclusions

We synthesized a molecular 5,10,15,20-tetrakis (4-ethylphenyl) porphyrin under mild and economic reaction conditions with a considerably higher reaction yield (over 50%) than in our previous report. Mixing pyrrole and 4-carboxybenzaldehyde in propionic acid to serve as a solvent and catalyst, respectively, followed by recrystallization with cold methanol and purification via column chromatography, was found to be an effective synthetic strategy. The chemical structure of the synthesized compound was verified using UV-Vis, FT-IR, and fluorescence techniques.

The 5,10,15,20-tetrakis (4-ethylphenyl) porphyrin showed adequate quantum yields of singlet oxygen and fluorescence (Φ_f_ = 0.12 ± 0.04 and Φ_Δ_ = 0.81 ± 0.23), which inhibited the growth of *P. aeruginosa*, *S. aureus*, and MRSA bacteria. In previous studies, irradiation for short periods was required to photoactivate porphyrin. In this study, photoactivation increased with the porphyrin concentration, resulting in higher bacterial mortality. The wildtype strain of *S. aureus* was sensitive to porphyrin treatment with and without irradiation. Thus, 5,10,15,20-tetrakis (4-ethylphenyl) porphyrin could be used to treat infections caused by susceptible strains of *S. aureus* where light may or may not be applied for porphyrin photoactivation.

## Figures and Tables

**Table 1:** MICs and calculated R^2^ values for treatment of the test bacteria with and without irradiation.

Bacterium	Treatment	MIC (µg/mL)	R^2^
*P. aeruginosa*	Irradiation	54.71	0.98
*P. aeruginosa*	No irradiation	402.9	0.98
*S. aureus*	Irradiation	67.68	0.92
*S. aureus*	No irradiation	58.26	0.99
MRSA	Irradiation	69.42	0.99
MRSA	No irradiation	109.9	0.91

## Data Availability

Data is contained within the article.
